# Awake Flexible Scope Intubation in Critically Ill Patients With Physiologically Difficult Airways: A Prospective, Observational Case Series

**DOI:** 10.1097/CCE.0000000000001394

**Published:** 2026-04-06

**Authors:** Daenis C. D. Camiré, Tara S. Ramaswamy, Lawrence F. Chu, Frederick G. Mihm

**Affiliations:** 1 Department of Anesthesiology and Perioperative Medicine, Kingston Health Sciences Centre, Kingston, ON, Canada.; 2 Department of Anesthesiology, Perioperative and Pain Medicine, Stanford University Medical Center, Stanford, CA.

**Keywords:** awake intubation, hypoxia, intubation complications, physiologically difficult airway

## Abstract

**IMPORTANCE AND OBJECTIVES:**

Asleep tracheal intubation is a potentially dangerous intervention in critically ill patients. Patients at high-risk of severe hypoxemia or hemodynamic collapse have a “physiologically difficult airway.” This study aimed to evaluate the procedural success and maintenance of physiologic stability associated with awake flexible scope intubation in these patients.

**DESIGN:**

Prospective single-center observational case series.

**SETTING:**

Tertiary critical care unit.

**PARTICIPANTS:**

Adults 18 years old or older requiring intubation who were considered to have a “physiologically difficult airway.”

**MAIN OUTCOMES AND MEASURES:**

All patients underwent awake intubation under topical anesthesia. The primary outcome was a composite endpoint consisting of first-pass success and the absence of serious physiologic events, defined as severe hypoxemia (pulse oximetry saturation [Spo_2_] < 80%), hypotension (mean arterial pressure [MAP] < 60 mm Hg), hypertension (MAP > 120 mm Hg), new arrhythmia, or cardiac arrest. Continuous recording of the bedside monitor, time points during intubation, and six aspects of cooperation were documented.

**RESULTS:**

All 39 patients had successful intubation on first attempt (100%). Of those fully studied, 25 of 36 (69.4%) met the composite endpoint: intubation plus physiologic stability. There were no arrhythmias or arrests. Spo_2_ decreased from baseline of 98% to 95% (*p* < 0.001) with one patient recording a Spo_2_ less than 80%. Peak heart rate increased from baseline of 95–105 beats/min (*p* < 0.001). MAP changed from baseline 87 mm Hg to lowest, 72 mm Hg (*p* < 0.001) and highest, 98 mm Hg (*p* < 0.001). Four patients had a recorded MAP less than 60 mm Hg (11%), and six patients had a MAP greater than 120 mm Hg (17%), but nine of the ten of these hemodynamic events lasted less than 30 seconds.

**CONCLUSIONS AND RELEVANCE:**

In critically ill patients with a physiologically difficult airway requiring intubation, awake flexible scope intubation may be an alternative to asleep tracheal intubation to help preserve hemodynamics. Further studies are needed to validate these findings.

**KEY POINTS**

**Question**: The aim of this study was to demonstrate the success and tolerance of awake flexible scope intubation in patients with a physiologically difficult airway.

**Findings**: All 39 patients had successful awake intubation on single attempt with topical anesthesia and careful coaching. Success was achieved without requiring complete cooperation in the majority of patients. Although some physiologic changes were observed, they were substantial in only a few patients and not clinically significant because of their brevity.

**Meaning**: Awake flexible scope intubation is a reasonable alternative to rapid-sequence induction techniques in patients with a physiologically difficult airway.

Tracheal intubation, usually performed with anesthetic induction (“asleep”), is a commonly performed and high-risk intervention in critically ill patients and is associated with major adverse peri-intubation events, including severe hypoxemia (pulse oximetry saturation [Spo_2_] < 80%), hypotension, and cardiac arrest (1–3). Patients who are at high-risk of developing hypoxemia or circulatory collapse peri-intubation are considered to have a “physiologically difficult airway” (PDA) (4–11). Current guidelines from a PDA Task Force recommend two “asleep” approaches for intubation: modified rapid-sequence induction (which includes small tidal volume mask ventilation) and delayed-sequence induction, which allows patients to maintain spontaneous ventilation as long as possible to optimize preoxygenation (10). Awake intubation is not commonly emphasized in these guidelines.

Awake flexible scope (previously called awake fiberoptic) intubation (AFSI) is a well-established approach for anticipated difficult airway management and is recommended by major airway societies because it preserves spontaneous ventilation and airway tone while allowing controlled, stepwise airway instrumentation under topical anesthesia (12–14). Importantly, this approach avoids the use of anesthetic induction agents, neuromuscular blockade, apnea, and the sudden onset of positive pressure ventilation, all of which can precipitate hypoxemia or hemodynamic collapse in physiologically vulnerable patients (1–3, 7, 10). In operating room settings, awake flexible scope techniques have high success rates with low severe adverse event rates when performed using structured topicalization protocols (14, 15). Despite increasing attention to the PDA concept in critical care, prospective ICU data describing physiologic tolerance and procedural outcomes during AFSI in patients considered to have a PDA remain limited, supporting the need for studies evaluating feasibility, success, and physiologic stability with an awake approach in this population. Our aim is to describe the use of AFSI in critically ill patients with a PDA.

## METHODS

After institutional review board (IRB) approval, we performed a prospective, observational case series to document outcomes of AFSI in critically ill patients with a PDA from January 2022 to June 2024 at Stanford Health Care (IRB No. 62415, November 1, 2021, Revision April 23, 2024). A Strengthening the Reporting of Observational Studies in Epidemiology checklist was used (**Supplemental File #1**, https://links.lww.com/CCX/B613). We followed procedures in accordance with the Helsinki Declaration of 1975. Patients were selected for AFSI when the attending intensivist determined that the patient had a PDA, defined as a high likelihood of clinically significant hypoxemia or hemodynamic decompensation following anesthetic induction, apnea, paralysis, and/or abrupt transition to positive pressure ventilation despite ongoing resuscitation and optimization (**Table 1**) (12, 13). As such, PDA designation was based on the severity and refractoriness of underlying physiology at the time of intubation decision-making. Specifically, AFSI was chosen in patients with features such as apnea intolerance with persistent high work of breathing or inadequate oxygenation despite high-flow oxygen or noninvasive ventilation, shock requiring vasoactive support in whom induction was judged likely to precipitate collapse, or cardiopulmonary conditions (i.e., pulmonary hypertension or right ventricular failure) where loss of spontaneous ventilation was considered particularly deleterious. Diagnoses such as intracranial hypertension, ventricular dysfunction, or aspiration risk were not considered indications for PDA in isolation but were contextual factors present in patients who also met physiologic risk criteria. Informed consent was obtained from a family member. Exclusion criteria included patients younger than 18 years old, pregnancy, patients in cardiac arrest, or those requiring immediate (“crash”) intubation. A “crash” intubation is an urgent intervention performed immediately to secure the airway during critical scenarios necessitating rapid stabilization or resuscitation, with little to no time for standard preparation or planning. The primary outcome was a composite endpoint consisting of first-pass success and the absence of serious events, defined as severe hypoxemia (Spo_2_ < 80%), hypotension (mean arterial pressure [MAP] < 60 mm Hg), hypertension (MAP > 120 mm Hg), new arrhythmia, or cardiac arrest. Continuous physiologic data were obtained directly from the bedside monitor, using a real-time recording device (Epiphan Pearl Nano device; Epiphan Video, Palo Alto, CA). Age, sex, medical conditions, vasoactive drugs, respiratory support, hemodynamics, Glasgow Coma Scale, Richmond Agitation-Sedation Scale, and Sequential Organ Failure Assessment (SOFA) before AFSI were recorded.

**TABLE 1.** Indicators of a Physiologically Difficult Airway

Acute respiratory failure^a^ requiring intubation, plus:
1) Severe shock^b^
2) Anterior mediastinal mass^c^
3) Right ventricular failure^d^
4) Severe pulmonary hypertension^e^
5) Mechanical assist devices^f^
6) Apnea intolerance, hypoxemia requiring high-flow oxygen therapy, bilevel positive pressure ventilation^g^
7) Severe metabolic acidosis^h^
8) Ischemic cerebrovascular accident/intracranial hemorrhage with risk of extension^i^
9) Patients with difficult airways who would be difficult to mask ventilate^j^
10) High aspiration risk^k^

a Life-threatening acute impairment of oxygenation (Pao_2_ < 60 mm Hg), carbon dioxide elimination (Paco_2_ > 45 mm Hg), or both on room air.

b A life-threatening condition characterized by a generalized, acute failure of circulation, where the delivery and use of oxygen by cells is insufficient, leading to multiple organ dysfunction, end-organ damage, and can result in death.

c Includes various neoplastic and non-neoplastic lesions in the anterior mediastinum that can cause life-threatening cardiovascular, respiratory, neurologic, endocrine complications, and/or superior vena cava syndrome.

d A clinical syndrome characterized by the presence of heart failure signs and symptoms that are attributable to right ventricular dysfunction, as demonstrated by abnormal structure or function leading to cardiogenic shock.

e Right ventricular systolic pressure or pulmonary artery systolic pressure > 90 mm Hg or a mean pulmonary artery pressure > 55 mm Hg.

f Right and/or left ventricular assist device and/or extracorporeal membrane oxygenation.

g Inability to withstand brief periods of breathing cessation without experiencing critical oxygen desaturation (< 80%).

h Anion gap and/or nonanion gap metabolic acidosis with a pH < 7.20.

i Critical, life-threatening intracranial hemorrhage occurring when there is high risk of increased bleeding volume if sudden and prolonged acute hypertension develops during tracheal intubation.

j Clinical situation where a skilled airway operator may experience difficulties with face mask ventilation or supraglottic airway, laryngoscopy, and/or tracheal intubation.

k Estimated based on “full stomach” status as per ASA fasting guidelines (i.e., recent cessation of large-volume feeds), active regurgitation or uncontrolled gastroesophageal reflux disease, decreased consciousness (Glasgow Coma Scale < 8), neurologic impairment or severe weakness compromising protective airway reflexes, and/or concurrent difficult airway features where face mask or supraglottic airway ventilation may result in high positive pressure (> 20 cm H_2_O), risking gastric insufflation, and pulmonary aspiration.

Physiologically difficult airway criteria were defined using physiologic thresholds indicating limited reserve or refractoriness to optimization. Listed conditions reflect clinical contexts in which induction, apnea, paralysis, or abrupt initiation of positive pressure ventilation was judged likely to cause decompensation despite resuscitation.

Secondary outcomes included peak MAP and heart rate (HR) change from the preprocedure baseline, peri-intubation vasopressor and sedation requirements, and total procedural time and patient cooperation.

An observer recorded six time points (min): start of topical anesthesia, placement of an intubating oral airway, start of AFSI, tracheal intubation, tracheal tube cuff inflation, initiation of positive pressure ventilation, and 5 minutes post-intubation. Six aspects of patient cooperation throughout the AFSI procedure were documented as either “yes” or “no” in response to verbal commands: opens mouth, gargles lidocaine, accepts oral airway, bites down on airway to hold it in place, and deep inspiration during local anesthetic spray and during tracheal tube advancement (“railroading”) into the trachea over the scope.

### Method of Flexible Scope Intubation

The procedure used topical anesthesia with 4% lidocaine administered with a malleable sprayer (MADgic laryngo-tracheal mucosal atomization device, MAD600; Teleflex, Morrisville, NC). Some initial topical anesthesia was used in the back of the mouth and then a “spray-as-you-go” technique was followed (14, 16). The topical spray was supplemented with 5% lidocaine ointment applied to the underside of the oral intubating airway (Hudson RCI Ovassapian Fiber Optic Intubating Airway; Teleflex). The amount of lidocaine used was well below the maximum dose of 9 mg/kg by lean body weight, following awake intubation guidelines (17). Lean body weight was calculated using the Boer formula (18). All patients were premedicated with IV glycopyrrolate 0.2–0.4 mg at least 10–20 minutes before topicalization to dry secretions, to improve visualization and local anesthetic contact. A 5.5 mm external diameter flexible scope (Storz 11301BNXK, Karl Storz SE & Co. KG; El Segundo, CA) was used for all intubations.

IV sedation (i.e., dexmedetomidine infusion), if present before intubation, was continued unchanged. No additional sedatives, induction agents or neuromuscular blockers were administered during AFSI. Additional analgesia (i.e., fentanyl IV) was generally avoided but assessed in each case. During the 5 minutes after AFSI, patients were assessed and analgosedation IV infusions were slowly started in anticipation of diminishing effects of the local anesthetic.

For intubation, all patients were in a “head-up” sitting position. AFSI was performed facing the patient. Patients not receiving ventilatory support were placed on high-flow oxygen therapy at 30–40 L/min at 100% Fio_2_ before AFSI. If patients were receiving bilevel positive pressure ventilation (BiPAP), the settings were unchanged except for exchanging the BiPAP mask for a modified Patil mask (Endoscopy Mask 30-40-555, VBM Medizintechnik GmbH; Sulz am Neckar, Germany) with 100% Fio_2_. The same topicalization and AFSI technique was employed whether high-flow oxygen or BiPAP was used. Additional oxygen therapy at 4 L/min through the working channel of the flexible scope helped keep secretions off the end of the scope but was low enough to allow for effective suctioning when needed. Pulmonary vasodilators (i.e., nitric oxide), if used, were continued throughout the intubation procedure.

Initial ventilator settings used a pressure support of 5–10 cm H_2_O and positive end-expiratory pressure of 5 cm H_2_O. Those receiving BiPAP were initially transitioned after intubation to identical pre-intubation ventilator settings. Patients were intubated with a 6.5–8.0 mm internal diameter oral tracheal tube (Shiley TaperGuard Oral Tracheal Tube 18870-18880; Medtronic, Minneapolis, MN).

The importance of avoiding “nocebo” language (verbal cues that raise negative expectations) and putting the patient at ease with nonthreatening instructions and comforting communication during the procedure cannot be overemphasized (**Supplemental File #2**, https://links.lww.com/CCX/B613) (19).

### Statistical Analysis

The composite primary outcome was defined at the patient level as a binary indicator of both procedural and physiologic success, reflecting real-world airway management priorities in the ICU. A patient met this outcome if first-pass intubation was achieved and no major physiologic events (Spo_2_ < 80%, MAP < 60 or > 120 mm Hg, new arrhythmia, or cardiac arrest) occurred. This composite endpoint was chosen because both technical success and physiologic stability are required for true clinical success in the critically ill. The proportion of patients meeting this endpoint was summarized with exact 95% binomial CIs. All analyses were performed using R, Version 4.1.0 (R Core Team, Vienna, Austria). Data normality was assessed using Shapiro-Wilk tests and Q-Q plots. For non-normally distributed data, results are presented as median (interquartile range [IQR]) and analyzed using nonparametric tests. Statistical significance was set at *p* value of less than 0.05, with Bonferroni correction applied for multiple comparisons. Primary outcomes were analyzed using paired nonparametric tests comparing baseline and peak/nadir values. Linear mixed-effects models were also applied to account for within-patient variation. These approaches were chosen to capture physiologic changes without assuming normal data distribution. The goal was to describe clinically relevant trends rather than emphasize statistical magnitude.

For subgroup analyses (i.e., cooperation, BiPAP, and vasopressors), mixed-effects models were used with random intercepts for patients. The chi-square tests assessed relationships between categorical variables. To evaluate within-subject changes in physiologic parameters (Spo_2_, MAP, and HR), we used linear mixed-effects models with random intercepts for each patient. Models were fit using the lmerTest package in R (Technical University of Denmark, DTU Compute, Kongens Lyngby, Denmark). Timepoint (baseline vs. during procedure) was included as a fixed effect.

Sample size was calculated to detect a median absolute decrease of 3% in Spo_2_ from baseline (i.e., 98% to 95%) with a sd of approximately 3.5%, based on prior peri-intubation hypoxia studies (1). Analysis based on the collected data showed an observed sd of 6.3%, yielding an achieved power of approximately 79.5% with 36 patients. Differences were evaluated within-patient with statistical analysis comparing baseline to nadir/peak values during continuous monitoring in each patient. This decision reflects clinical practice, focusing on worst-case physiologic changes. A data analysis and statistical plan was written after data collection but before final analysis.

## RESULTS

### Patient Characteristics and Procedural Success

Thirty-nine consecutive patients (mean = 42 yr old, sd 15, 67% male) were enrolled in the study. All 39 patients (100%) had successful AFSI on first attempt. Three patients were excluded from further analysis because the physiologic recording was not obtained or corrupted by human error. Thirty-six patients were analyzed for physiologic data and 25 (69.4%; 95% CI, 52.9–83.7%) met our strict composite endpoint (successful intubation without hypoxemia, hemodynamic instability, new arrhythmia, and/or cardiac arrest). The most common reason for failure to reach this endpoint was transient hypertension, which occurred in six of 11 patients (55%).

**Figure 1.**
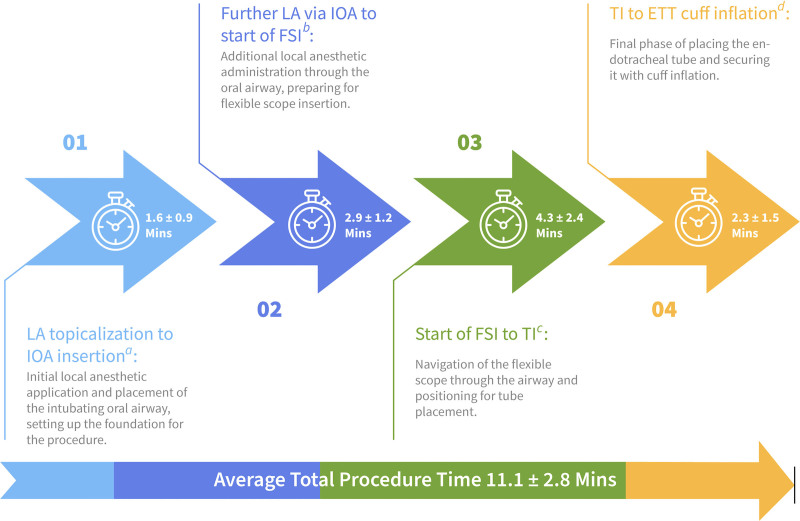
Timeline of the awake flexible scope intubation (FSI) procedure. Timeline of the awake FSI procedure (min; i.e., mean time at each procedural step ± sd) from the start of local anesthetic (LA) topicalization to endotracheal tube (ETT) cuff inflation. ^a^LA, intubating oral airway (IOA). ^b^FSI. ^c^Tracheal intubation (TI), that is, “railroading” of the ETT over the flexible scope into the trachea. ^d^ETT.

Patient characteristics are recorded in **Table 2**. Most patients (70%, 25/36) had three or more concurrent physiologically vulnerable conditions. Coexisting high-risk factors included increased aspiration risk, intracranial hypertension and difficult airway features. The mean time (including topicalization) for these awake intubations was 11.1 minutes (**Fig. 1**). Most procedures (81%, 29/36) were performed by Critical Care Medicine fellows from various backgrounds (pulmonary medicine, neurology, internal medicine, infectious disease, nephrology, cardiology, surgery, emergency medicine) and anesthesia residents, with 67% (24/36) of operators having less than five previous AFSI attempts and 25% (9/36) having no previous experience. Trainees were always supervised by a single experienced anesthesiologist intensivist.

**TABLE 2.** Clinical Features of the 36 Study Patients

Pre-Intubation Characteristics		Comorbidities, *n* (%)		Peri-Intubation Events, *n* (%)	
Indication for intubation, *n* (%)		Airway and respiratory, *n* (%)		During intubation, *n* (%)	
Hypoxemia^a^	19 (53)	Aspiration risk^k^	23 (64)	Vasopressor increase	0 (0)
Hypercarbia^b^	11 (31)	Difficult airway^l^	8 (22)	Vasopressor decrease	0 (0)
Mixed respiratory failure^c^	3 (8)	Anterior mediastinal mass^m^	4 (11)	Vasodilator increase	0 (0)
Altered mental status (Glasgow Coma Scale ≤ 9)	5 (14)	Pulmonary embolism	2 (6)	Vasodilator decrease	0 (0)
Airway obstruction^d^	2 (6)	Heart failure		Sedative bolus	0 (0)
Severity of illness, median (interquartile range)		Biventricular^n^	8 (22)	Analgesic bolus	4 (11)
Sequential Organ Failure Assessment score	8.5 (4–10)	Left ventricular^o^	8 (22)	Post-Intubation	
Shock and etiology, *n* (%)		Right ventricular^p^	3 (8)	Vasopressor increase	2 (6)
Presence of shock	18 (50)	Left ventricular assist device	1 (3)	Vasopressor decrease	2 (6)
Distributive^e^	7 (19)	Severe valvulopathy^q^		Vasodilator increase	0 (0)
Cardiogenic^f^	4 (17)	Mitral stenosis	1 (3)	Vasodilator decrease	1 (3)
Undifferentiated^g^	3 (8)	Aortic stenosis	1 (3)	Sedative bolus^t^	1 (8)
Obstructive^h^	0 (0)	Pulmonary hypertension^r^		Analgesic bolus	7 (19)
Mixed^i^	4 (11)	Mild	8 (22)		
Cardiogenic and distributive	3 (8)	Moderate	2 (6)		
Cardiogenic and hypovolemic^j^	1 (3)	Severe	2 (6)		
Vasopressors infusions, *n* (%)		Neurologic			
Any vasopressor	18 (50)	Intracranial hemorrhage^s^	3 (8)		
1 Vasopressor	8 (22)				
2 Vasopressors	2 (6)				
3 Vasopressors	8 (22)				
Vasodilator infusions, *n* (%)					
1 Vasodilator	2 (6)				
Sedative and analgesics, *n* (%)					
1 Sedative infusion	3 (8)				
1 Analgesic bolus	4 (11)				

a Acute impairment of oxygenation (Pao_2_ < 60 mm Hg) on room air.

b Acute impairment of carbon dioxide elimination (Paco_2_) > 45 mm Hg) on room air.

c Acute impairment of oxygenation and carbon dioxide elimination (Pao_2_ < 60 mm Hg and Paco_2_ > 45 mm Hg).

d Acute stridor and respiratory distress causing severe respiratory compromise or impending respiratory failure.

e Severe peripheral vasodilatation and reduced systemic vascular resistance, often with normal or increased cardiac output.

f Primary cardiac failure causing end-organ hypoperfusion.

g Cardiocirculatory failure characterized by end-organ hypoperfusion, with an unidentified underlying etiology.

h Cardiac failure from noncardiac diseases causing end-organ hypoperfusion.

i End-organ hypoperfusion with at least two shock types present, causing complex and sometimes conflicting hemodynamics.

j Hemorrhagic or nonhemorrhagic resulting in reduced intravascular volume, cardiac output, and end-organ hypoperfusion.

k No patient was intubated solely due to aspiration risk. Estimated based on “full stomach” status as per ASA fasting guidelines (i.e., recent cessation of large-volume feeds), active regurgitation or uncontrolled gastroesophageal reflux disease, decreased consciousness (Glasgow Coma Scale < 8), neurologic impairment or severe weakness compromising protective airway reflexes, and/or concurrent difficult airway features where face mask or supraglottic airway ventilation may be inadequate and result in high amounts of positive pressure (> 20 cm H_2_O), risk gastric insufflation and pulmonary aspiration.

l Clinical situation where a skilled airway operator may experience difficulties with face mask or supraglottic airway ventilation, laryngoscopy, and/or tracheal intubation, involving challenges like inadequate oxygenation, ventilation, or visualization of the vocal cords after multiple attempts.

m Includes various neoplastic and non-neoplastic lesions in the anterior mediastinum that can cause life-threatening cardiovascular, respiratory, neurologic, endocrine complications, and/or superior vena cava syndrome.

n Presence of heart failure signs and symptoms that are attributable to left and right ventricular dysfunction, as demonstrated by abnormal structure or function leading to cardiogenic shock.

o Left ventricular dysfunction, as demonstrated by abnormal structure or function leading to cardiogenic shock.

p Right ventricular dysfunction, as demonstrated by abnormal structure or function leading to cardiogenic shock.

q Moderate-to-severe stenosis or regurgitation with hemodynamic consequences and/or symptoms.

r Right ventricular systolic pressure: mild (40–60 mm Hg), moderate (60–90 mm Hg), and severe (> 90 mm Hg); mean pulmonary artery pressure: mild (25–40 mm Hg), moderate (41–55 mm Hg), and severe (> 55 mm Hg).

s Ongoing or a high risk of increased bleeding volume, which can worsen if sudden and prolonged acute hypertension develops from tracheal intubation.

t Propofol bolus given by a clinician unfamiliar with the study protocol in one patient.

### Preprocedure Status

Preprocedural consciousness was variable (median Glasgow Coma Scale = 14 [IQR, 13–15; range, 8–15], Richmond Agitation-Sedation Scale = –3 to +2; **Table 3**). Respiratory support included 3 (8%) with nasal cannula, 3 (8%) nonrebreather mask, 12 (33%) high-flow nasal cannula or combined with a nonrebreather mask, and 15 (42%) BiPAP, with 19 (53%) requiring 100% Fio_2_, including five patients (14%) receiving inhaled pulmonary vasodilators (i.e., nitric oxide at 20 parts per million) pre-intubation. Fifty percent of patients (50%) required vasopressors before intubation and the cohort exhibited a median SOFA score (8.5 [IQR, 4–10]).

**TABLE 3.**
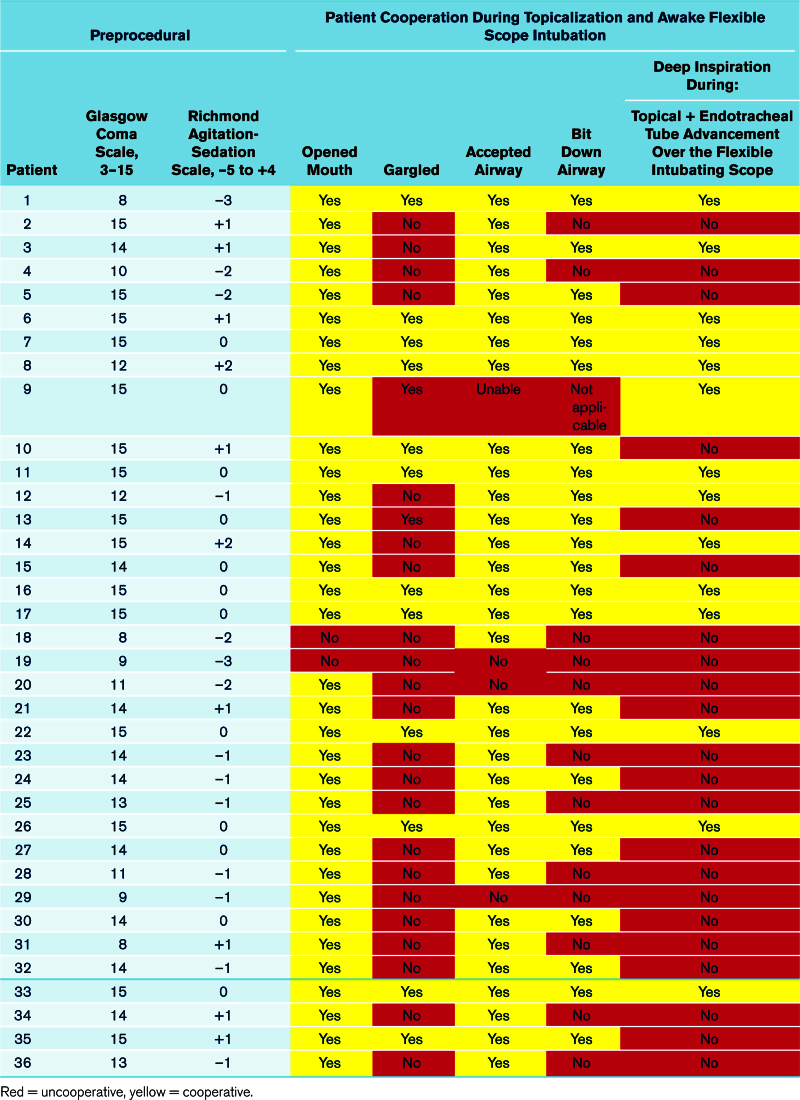
Preprocedural and Peritracheal Intubation Level of Cooperation

### Vital Sign Changes and Cooperation

**Figure 2.**
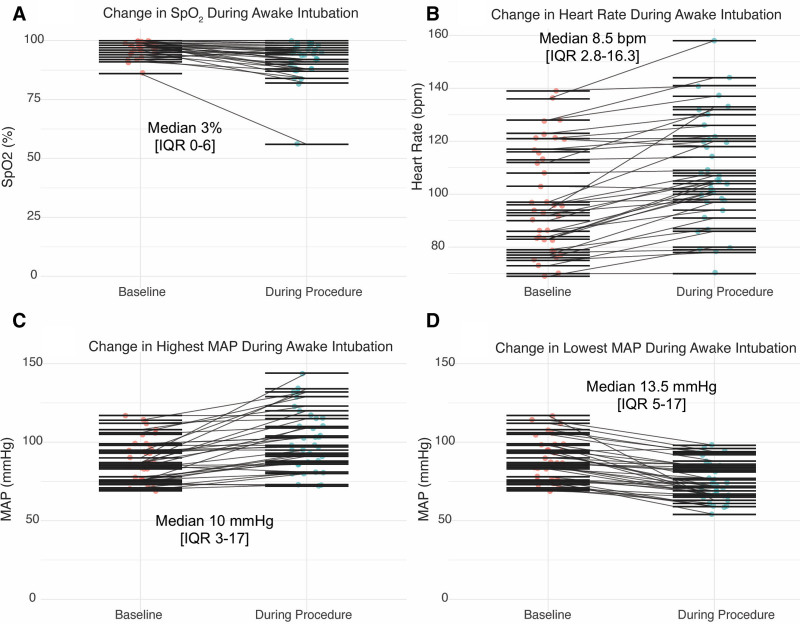
Physiologic changes during awake intubation. *Paired plots* show individual patient trajectories from baseline (*red dots*) to peak value (*blue dots*) during the procedure for oxygen saturation (Spo_2_) (**A**), heart rate (HR) (**B**), highest mean arterial pressure (MAP) (**C**), and lowest MAP (**D**). *Each line* represents a single patient. Annotated values represent the median within-patient change with interquartile range (IQR). Median Spo_2_ decreased by 3% (IQR, 0–6%), HR increased by 8.5 beats/min (bpm; IQR, 2.8–16.3 bpm), highest MAP increased by 10 mm Hg (IQR, 3–17 mm Hg), and lowest MAP decreased by 13.5 mm Hg (IQR, 5–17 mm Hg).

Individual paired patient physiologic responses during and 5 minutes after AFSI are shown in **Figure 2** and **Supplemental File #3** (https://links.lww.com/CCX/B613). An arterial line was present in 30 patients (83%). During awake intubation, patients experienced modest but consistent physiologic changes.

Median baseline Spo_2_ was 98% (IQR, 96–99%), and median lowest Spo_2_ during the procedure was 95% (IQR, 89.5–98%), corresponding to a median within-patient decrease of three percentage points (IQR, 0–6; Wilcoxon signed-rank test, V = 383; *p* < 0.001). One patient (3%, 1/36) who had a baseline Spo_2_ of 86%, experienced Spo_2_ less than 80% that occurred after the airway was secured, associated with a delay in switching from high flow oxygen to mechanical ventilation. This desaturation lasted 119 seconds.

Median baseline HR was 95 beats/min (bpm; IQR, 83–116 bpm), and median highest HR during the procedure was 105 bpm (IQR, 98–121 bpm), corresponding to a median within-patient increase of 8.5 bpm (IQR, 2.8–16.2 bpm; Wilcoxon signed-rank test, V = 10.5; *p* < 0.001). This represents a 9% change in HR overall, with eight patients showing less than or equal to 0–1% HR change. Baseline HR reflected any changes induced by glycopyrrolate.

MAP demonstrated bidirectional changes, with a median baseline MAP of 87 mm Hg (IQR, 76–95.8 mm Hg). Median lowest MAP during the procedure was 72 mm Hg (IQR, 66–84 mm Hg), corresponding to a median within-patient decrease of 13.5 mm Hg (IQR, 5–17.2 mm Hg; Wilcoxon signed-rank test, V = 627; *p* < 0.001). Median highest MAP during the procedure was 98 mm Hg (IQR, 86.8–115 mm Hg), corresponding to a median within-patient increase of 10 mm Hg (IQR, 3–17.2 mm Hg; Wilcoxon signed-rank test, V = 11; *p* < 0.001). Four patients (11%, 4/36) experienced a MAP less than 60 mm Hg and six (17%, 6/36) had a MAP greater than 120 mm Hg, with the highest being 144 mm Hg. All hemodynamic events were brief (in 9/10 events, duration < 30 s except one episode lasting 72 s) and without consequence. These observations represent a 15 mm Hg (17%) decrease and a 14 mm Hg (16%) increase from baseline MAP. No patients experienced cardiac arrhythmias or arrest. Only two of 36 patients (6%) required any increase in vasopressor infusion during this procedure and this occurred from an “off protocol” sedative bolus in one of them. To summarize these findings, small yet statistically significant changes in Spo_2_, HR, and MAP were observed. However, these shifts, when large, were brief and clinically minor, resolving spontaneously in all cases. Mixed-effects models confirmed that patient cooperation, ventilatory mode, and vasopressor use did not meaningfully influence these physiologic trends. These findings reinforce that AFSI maintains hemodynamic and oxygenation stability independent of these variables.

Patient cooperation during AFSI was highly variable, with only 28% (10/36) able to follow all six instructions, 94% (34/36) opened mouth, 39% (14/36) gargled, 89% (32/36) accepted airway, 64% (23/36) bit down airway, and 39% (14/36) coordinated deep inspiration with anesthetic spraying and advancement of the tracheal tube over the scope (Table 3). Airway topicalization was performed in all patients regardless of mental status to minimize airway reflex activation and reduce the need for systemic sedation. No patients received more than 400 mg of lidocaine and smaller patients (≤ 55 kg) received 300 mg with no cases of toxicity observed.

Three patients had preexisting sedative infusions, which were not adjusted during intubation. Aliquots of fentanyl 12.5–25 µg IV (maximum dose 100 µg) were administered during tracheal tube advancement or post-intubation in some patients. After intubation, patients remained calm and were reassured by providers.

## DISCUSSION

### Principal Findings and Clinical Significance

In our case series of critically ill patients with a PDA, AFSI achieved 100% first-pass success. Nearly 70% of patients achieved the composite outcome, first-pass success without severe physiologic disturbance, which we deliberately defined to mirror real clinical judgment. By combining procedural and physiologic endpoints, we captured patient-centered safety rather than procedural completion alone. In large prospective “asleep” intubation cohorts such as the INTUBE study, approximately 80% of intubations are successful on the first attempt; however, more than 40% of the successful intubations lead to severe physiologic events, highlighting the risks of emergency intubation in the ICU (1–3, 20, 21). We noted fewer serious events and no cases of new-onset arrhythmia or cardiac arrest compared with recent “asleep” intubation studies reporting rates of cardiovascular instability (42.6%) requiring initiation or escalation of vasopressors (87.8%), severe hypoxemia (20.3%), new-onset arrhythmia (5.6%), and cardiac arrest (3.1%) (1–3, 20, 21). Of these arrests, 47% do not achieve return of spontaneous circulation (1, 20, 22, 23). Any peri-intubation event is an independent risk factor for 28-day mortality, with rates reaching up to 39.5% compared with 28.5% in the absence of such events (2, 20, 22). Cardiac arrest is associated with a 14-fold increased odds of in-hospital mortality, with intubation survivors experiencing mortality rates as high as 82% (21).

Many of the physiologic changes that we documented would not have been detected with standard vital signs measured at 5-minute intervals; however, we think these were not clinically important because of their brevity (< 30 s). Any break past our stated thresholds was counted as an “event,” no matter how brief. Future studies should employ less stringent and time-based criteria (i.e., MAP < 60 mm Hg for > 1–2 min). Clinically significant periods of hypotension and hypoxemia (MAP < 65 mm Hg and Spo_2_ < 80% sustained for 30 min or longer) have been reported in “asleep” intubation studies using similar methods (1–3, 20, 22). Importantly, success in our study was achieved without reliance on systemic sedation and without total cooperation in many patients, challenging common perceptions surrounding “awake” intubation requirements.

A significant component of our success was the avoidance of nocebo language. This refers to words or phrases that unintentionally increase negative expectations leading to worse outcomes for patients (24) (Supplemental File #2, https://links.lww.com/CCX/B613).

Physiologic deterioration during intubation in critically ill patients is multifactorial, often stemming from underlying disease processes such as hypoxemia, hypercarbia, circulatory shock, intracranial hypertension, and the initiation of positive pressure ventilation. However, the administration of sedatives, induction agents, neuromuscular blockers, and subsequent apnea can represent a significant additional source of respiratory and hemodynamic instability (1–3, 7, 20, 25). The subset of ICU patients with a PDA would be expected to have an even higher incidence of complications. These patients are challenging, as they exhibit limited preoxygenation effectiveness and exaggerated negative hemodynamic responses to traditional rapid-sequence induction methods (5, 7, 10). While current guidelines primarily recommend “asleep” approaches (10, 11, 26), our findings suggest an effective alternative “awake” strategy. Although some physiologic parameters reached statistical significance in our study, their magnitude and duration were small, underscoring that AFSI preserves stability even in high-risk patients. The statistical analyses served mainly to confirm what was evident clinically: the procedure is both effective and well tolerated.

### Evolution of Physiologically Difficult Airway Management

The PDA concept was first described using the Consumption increase, Right ventricular failure, Acidosis, decreased saturation, and Hypotension (CRASH) mnemonic and has expanded considerably (4, 27). Current indications include severe metabolic acidosis with hypotension, valvular heart disease, ventricular failure, pulmonary hypertension, severe refractory hypoxemia, Gram-negative septic shock, and patients prone to rapid desaturation (8, 9, 11, 15, 28–30). Recent additions encompass intracranial hypertension, pediatric populations, patients at risk of aspiration, and physiologic derangements of obesity and pregnancy (4, 7, 10, 11).

### Comparison With Current Evidence and Alternative Approaches

While AFSI demonstrates excellent success and safety in operating room settings (i.e., failure rate 0.59%, severe adverse events 0.34%) (17, 31) and is recommended for anatomically difficult airways (12, 13, 17), its role in PDA management continues to evolve. The Canadian Airway Focus Group, and the ASA difficult airway algorithms recommend “awake” approaches primarily for desaturation risk when abnormal airway anatomy is present (7, 8, 12, 13, 29), while the Society of Airway Management, Society of Critical Care Medicine, and the Society of Critical Care Anesthesiologists’ guidelines favor “asleep” tracheal intubation techniques (10, 11, 26).

Recent studies of “awake” videolaryngoscopy show promise but rely on heavy sedation. One study demonstrated reduced adverse events compared with “asleep” rapid-sequence induction (2.2% vs. 39%) but required high-dose dexmedetomidine (32), which many critically ill patients tolerate poorly (33). Previous flexible scope ICU intubation studies were retrospective with limited physiologic data. They also frequently depended on moderate to heavy sedation (24, 34–38), with one study reporting a 50% incidence of severe hypotension (systolic blood pressure < 70 mm Hg) (35).

### Methodological Strengths

The patients in our study represented a very sick study population, as reflected in our SOFA scores and 50% incidence of vasopressors needed before intubation. Other large ICU intubation studies reported only a 20–30% incidence of pre-intubation vasopressors (20, 23, 39, 40). Pre-intubation vasopressor requirement represents the highest risk factor for major cardiovascular collapse in these patients (20). Our median SOFA scores were also more than two points higher (8.5 vs. 6) than two large ICU intubation studies (20, 23). Our study uniquely employed prospective continuous physiologic recording. We also systematically documented patient cooperation beyond a superficial single word assessment (35), while maintaining true “awake” status aligned with some expert recommendations (12, 13, 17, 31), as the majority of our patients had a near normal level of consciousness.

Our AFSI technique demonstrated remarkable versatility across respiratory support modalities (42% on BiPAP, 53% requiring 100% Fio_2_) and a spectrum of cooperation levels. We incorporated elements of the Spontaneous, Awake Ventilation, Inotropic support, and Optimized Respiratory physiology (SAVIOR) algorithm (41), which emphasizes gradual positive pressure transitioning. This approach resulted in consistent physiologic stability. We included high MAP (> 120 mm Hg) criteria because the PDA definition is expanding to include conditions like intracranial hemorrhage, aortic dissection, etc, where sustained blood pressure elevations can be dangerous.

Most of our intubations were performed by trainees with limited prior AFSI experience under direct supervision, suggesting that, within a structured protocol emphasizing topicalization and communication, AFSI may be teachable and reproducible in ICU practice when expert oversight and appropriate preparation are present. This finding directly addresses a common critique of awake intubation—that it requires rare expertise—and suggests that AFSI may be more generalizable than previously assumed given that airway experts are available at all major institutions.

### Limitations and Future Directions

Our single-center study requires validation in larger cohorts, particularly for underrepresented PDA subgroups like severe pulmonary hypertension and pregnancy. Selection bias was possibly introduced based on the senior author’s availability for every intubation. Bias also may have occurred because the ICU team used clinical judgment to identify patients as high risk for an asleep intubation technique without prespecified objective thresholds. This limits reproducibility and comparability with other study populations. There were also missed opportunities, as an awake intubation was often not considered by the ICU team. However, the study captured a wide array of critically ill patients that are representative of real-world clinical practice and these patients conformed to the current definition of a PDA.

Observer bias risk was minimized by using real-time continuous data collection methods. Definition refinement (i.e., disease severity scores) may lead to better patient selection for future studies. We did not prospectively define or systematically capture awake-specific airway complications (i.e., aspiration/regurgitation, laryngospasm/bronchospasm, and airway trauma) but did document the incidence of conversion to an asleep technique, of which there were none. However, review of procedural and clinical documentation revealed no awake-specific complications, supporting the overall procedural safety of AFSI in this study. Finally, the “art” of AFSI—encompassing patient communication and topical anesthesia technique—remains difficult to quantify scientifically. This study should be considered exploratory, with findings intended to generate hypotheses for larger, multicenter validation studies.

Our results have limited applicability to patients requiring immediate (“crash”) tracheal intubation. The inability to include these patients skewed the population that we report, as in other studies. However, with airway equipment readily available, we found that we could respond to urgent intubation scenarios. ICUs must have dedicated equipment for this purpose so that setup time does not preclude this airway approach (25). It should also be noted that our study was not designed to demonstrate speed of intubation given that inexperienced operators were involved, but even so, we believe that 11 minutes (from start of topicalization to intubation) is shorter than the general perception about the time needed for awake intubations.

## IMPLEMENTATION STRATEGIES

Based on our findings, successful AFSI implementation requires:

1) Structured training by expert clinicians emphasizing topical anesthesia and communication skills.
2) Avoidance of systemic anesthetics to compensate for inadequate topical anesthesia or cooperation.
3) Standardized equipment sets (i.e., “go-bags”) immediately available to minimize preparation time.
4) Systematic team education focusing on preparation efficiency and task assignments, skilled supervisors, and simulation (**Supplemental File #4**, https://links.lww.com/CCX/B613).
5) Gradual ventilator transition protocols starting with low initial pressure support ventilator settings (41).

Our study demonstrates that AFSI is a feasible alternative in expert hands for intubations in critically ill patients who are at high-risk of complications during traditional “asleep” intubation methods that use general anesthesia, paralysis, apnea and the sudden onset of positive pressure ventilation. We had three key findings. First, we achieved 100% first-pass success even with inexperienced bronchoscopists but always under expert supervision. Second, we showed that with careful topical anesthetic technique and communication, AFSI can maintain acceptable vital signs and patient comfort. Third, we demonstrated that patients do not need to be fully cooperative or heavily sedated for the procedure to succeed. When performed by skilled practitioners, AFSI is a valid technique that compliments other airway management strategies in the ICU and may be particularly suitable for the high-risk patient.

## ACKNOWLEDGMENTS

We thank Dr. David Stahl and Debbie Mihm for proofreading our article before submission.

## Supplementary Material
