# Supplementary material for: Awake Flexible Scope Intubation in Critically Ill Patients With Physiologically Difficult Airways: A Prospective, Observational Case Series
Source: Crit Care Explor. 2026 Apr 6;8(4):e1394. doi: 10.1097/CCE.0000000000001394 (PMC13056465; doi:10.1097/CCE.0000000000001394)
Supplement: Supplementary file 1 [file cc9-8-e1394-s001.pdf]

SUPPLEMENTAL MATERIAL TABLE OF CONTENTS

1. **STROBE Statement** **pg 2-3**
2. **Communication Approach During Awake Flexible Scope Intubation (AFSI)**: **pg 4**
3. **Peri-Tracheal Intubation Hemodynamic and Oxygenation Related Outcomes** **pg 5**
4. **Setup for Awake Flexible Scope Intubation (AFSI)** **pg 6-8**

SUPPLEMENTAL FILE. #1

STROBE Statement—checklist of items that should be included in reports of observational studies

	**Item No**	**Recommendation**	**Page No**
**Title and abstract**	1	(*a*) Indicate the study's design with a commonly used term in the title or the abstract	1
		(*b*) Provide in the abstract an informative and balanced summary of what was done and what was found	2 – 3
**Introduction**			
Background/ rationale	2	Explain the scientific background and rationale for the investigation being reported	5
Objectives	3	State specific objectives, including any prespecified hypotheses	5
**Methods**			
Study design	4	Present key elements of study design early in the paper	5 – 7
Setting	5	Describe the setting, locations, and relevant dates, including periods of recruitment, exposure, follow-up, and data collection	5 – 6
Participants	6	(*a*) *Cohort study*—Give the eligibility criteria, and the sources and methods of selection of participants. Describe methods of follow-up<br>*Case-control study*—Give the eligibility criteria, and the sources and methods of case ascertainment and control selection. Give the rationale for the choice of cases and controls<br>*Cross-sectional study*—Give the eligibility criteria, and the sources and methods of selection of participants	5 – 6
		(*b*) *Cohort study*—For matched studies, give matching criteria and number of exposed and unexposed<br>*Case-control study*—For matched studies, give matching criteria and the number of controls per case	N/A
Variables	7	Clearly define all outcomes, exposures, predictors, potential confounders, and effect modifiers. Give diagnostic criteria, if applicable	5-6

Data sources/ measurement	8	For each variable of interest, give sources of data and details of methods of assessment (measurement). Describe comparability of assessment methods if there is more than one group	5– 7
Bias	9	Describe any efforts to address potential sources of bias	7
Study size	10	Explain how the study size was arrived at	9
Quantitative variables	11	Explain how quantitative variables were handled in the analyses. If applicable, describe which groupings were chosen and why	9
Statistical methods	12	(*a*) Describe all statistical methods, including those used to control for confounding	9
		(*b*) Describe any methods used to examine subgroups and interactions	9
		(*c*) Explain how missing data were addressed	9
		(*d*) *Cohort study*—If applicable, explain how loss to follow-up was addressed<br>*Case-control study*—If applicable, explain how matching of cases and controls was addressed<br>*Cross-sectional study*—If applicable, describe analytical methods taking account of sampling strategy	N/A
		(*e*) Describe any sensitivity analyses	9
**Results**			
Participants	13	(a) Report numbers of individuals at each stage of study—eg numbers potentially eligible, examined for eligibility, confirmed eligible, included in the study, completing follow-up, and analysed	9
		(b) Give reasons for non-participation at each stage	9
		(c) Consider use of a flow diagram	N/A
Descriptive data	14	(a) Give characteristics of study participants (eg demographic, clinical, social) and information on exposures and potential confounders	9 – 10
		(b) Indicate number of participants with missing data for each variable of interest	9
		(c) *Cohort study*—Summarise follow-up time (eg, average and total amount)	N/A
Outcome data	15	*Cohort study*—Report numbers of outcome events or summary measures over time	10 – 12
		Case-control study—Report numbers in each exposure category, or summary measures of exposure	N/A
		Cross-sectional study—Report numbers of outcome events or summary measures	N/A
Main results	16	(*a*) Give unadjusted estimates and, if applicable, confounder-adjusted estimates and their precision (eg, 95% confidence interval). Make clear which confounders were adjusted for and why they were included	9 – 12
		(*b*) Report category boundaries when continuous variables were categorized	10
		(*c*) If relevant, consider translating estimates of relative risk into absolute risk for a meaningful time period	N/A
Other analyses	17	Report other analyses done—eg analyses of subgroups and interactions, and sensitivity analyses	12
**Discussion**			
Key results	18	Summarise key results with reference to study objectives	12 – 14
Limitations	19	Discuss limitations of the study, taking into account sources of potential bias or imprecision. Discuss both direction and magnitude of any potential bias	15
Interpretation	20	Give a cautious overall interpretation of results considering objectives, limitations, multiplicity of analyses, results from similar studies, and other relevant evidence	16
Generalisability	21	Discuss the generalisability (external validity) of the study results	16

Other information			
Funding	22	Give the source of funding and the role of the funders for the present study and, if applicable, for the original study on which the present article is based	1

SUPPLEMENTAL FILE. #2

Communication Approach During Awake Flexible Scope Intubation (AFSI):

1. **Explain:** "I am here to help you. We are going to help you breathe. In order to do that we will need to place a breathing tube to connect you to the breathing machine. I am going to do this in the safest possible way for you. I am going to walk you through this, and you will be fine. Once we have the breathing tube in place, the breathing machine will help you breathe, and you can get some rest. The first step is to numb your mouth and windpipe with local anesthetic by simply gargling and breathing in the anesthetic."
2. **Gargle 4% Lidocaine**: "We are going to begin by spraying some local anesthetic in the back of your mouth. Try not to swallow this, but let it sit in the back of your mouth and gargle it if you can. Gargle, but do not swallow." **Spray 2 ml of 4% Lidocaine in the back of mouth.**
3. **Intubating Oral Airway (IOA)**: "Open your mouth. I'm placing an airway gently in your mouth that will help us place the breathing tube." **Place the IOA** (Hudson RCI® Ovassapian Fiber Optic Intubating Airway, Teleflex, Morrisville, NC, USA) **with 5% Lidocaine ointment spread thinly on the underside onto the posterior tongue.**
4. **Pulsed spray 4% Lidocaine**: "I am going to spray this local anesthetic while you breathe. Imagine that you are in the shower and just breathe in the mist. Take some nice big breaths." **Spray with each inspiration (2 – 4 ml total).**
5. **Advance Flexible Intubating Scope (FIS) into airway:** "Just close your eyes and concentrate on your breathing. I am just passing a little camera into your mouth to look into your airway." **Stop in front of vocal cords and spray 1 – 2 ml of 4% Lidocaine. Wait 20 seconds.**
   If they cough with additional local anesthetic: "That's just some more anesthetic numbing your airway. Everything is fine."
6. **Advance FIS into mid-trachea:** **Spray 1 – 2 ml of 4% Lidocaine. Wait 20 seconds.**
7. **Advance FIS to just in front of carina:** **Spray 1 – 2 ml of 4% Lidocaine. Wait 20 seconds.**
8. **Remove Ovassapian IOA before 'railroading' endotracheal tube (ETT) into airway:**
   "Open your mouth." "We don't need that oral airway anymore because you are doing so well." **Remove Ovassapian IOA.**
9. **'Railroad' ETT over FIS**: **ETT tip pointing toward anterior commissure and advance incrementally, ONLY on inspiration.** "Concentrate on your breathing and take nice big breaths in and out."
10. **Confirm position of ETT:** (Aim for 2 – 3 cm above the carina) **Technique: Position FIS at the level of the carina, pinch FIS just as it exits the ETT with thumb and index finger, keeping these fingers fixed on the scope, slowly withdraw the scope until the tip of the distal end of the ETT is visible and stop. Note the distance of your thumb and index finger from the proximal end of ETT = ETT tip to carina distance.**
11. **Start Pressure Support Ventilation/Peek End-Expiratory Pressure First:** (5/5 - 10/5 cmH_2O or same settings as Bilevel Positive Pressure Ventilation or Continuous Positive Airway Pressure if required pre-intubation)

12. **THEN inflate the ETT cuff**: "Everything went fine. You can rest now; we are helping you with your breathing."

SUPPLEMENTAL FILE #3: Peri-Tracheal Intubation Hemodynamic and Oxygenation Related Outcomes

Patient	SpO_2 Baseline (%)	Lowest SpO_2 (%)	MAP Baseline (mmHg)	Lowest MAP (mmHg)	MAP High (mmHg)	MAP Change from Baseline (%)	HR Baseline (bpm)	HR High (bpm)	HR Change from Baseline (%)
**1**	97	94	93	71	98	- 22 to + 5	128	137	+ 7
**2**	91	87	99	83	103	- 16 to + 4	96	104	+ 8
**3**	96	82	87	82	134	- 6 to + 35	83	101	+ 18
**4**	99	88	83	66	95	- 20 to + 13	123	130	+ 5
**5**	98	96	112	94	115	- 16 to + 3	136	158	+ 14
**6**	96	94	78	67	87	- 14 to + 10	112	132	+ 19
**7**	93	95	95	84	95	- 12 to + 0	103	102	- 1
**8**	98	87	94	96	132	+ 2 to + 29	69	80	+ 14
**9**	98	95	117	63	117	- 46 to + 0	83	104	+ 20
**10**	100	99	105	88	110	- 16 to + 5	128	144	+ 11
**11**	99	99	108	92	123	- 15 to + 12	77	97	+ 21
**12**	95	90	90	85	144	- 6 to + 37	121	126	+ 4
**13**	98	88	74	74	91	- 0 to + 19	86	101	+ 15
**14**	97	96	86	84	120	- 2 to + 28	117	122	+ 4
**15**	100	100	71	66	81	- 7 to + 18	121	118	- 3
**16**	99	96	114	98	132	- 14 to + 14	96	108	+ 11
**17**	100	91	99	66	103	- 33 to + 4	94	133	+ 29
**18**	99	84	84	72	109	- 14 to + 13	84	100	+ 16
**19**	94	88	75	52	110	- 31 to + 32	76	91	+ 16
**20**	100	99	87	86	115	- 1 to + 24	86	94	+ 9
**21**	86	56	98	76	98	- 22 to + 0	139	141	+ 1
**22**	99	99	69	65	80	- 6 to + 14	108	109	+ 1
**23**	98	97	76	69	80	- 9 to + 5	83	105	+ 21
**24**	98	98	76	59	93	- 22 to + 18	93	98	+ 5
**25**	98	92	73	59	73	- 19 to + 0	75	78	+ 4
**26**	95	95	106	93	129	- 12 to + 18	90	107	+ 16
**27**	92	97	70	61	73	- 13 to + 16	121	121	+ 0
**28**	92	84	76	71	88	- 7 to + 14	78	79	+ 1
**29**	100	100	93	72	103	- 13 to + 10	92	105	+ 12
**30**	100	95	85	69	81	- 19 to + 5	97	98	+ 1
**31**	98	92	95	66	97	- 31 to + 2	113	114	+ 1

**32**	96	92	74	59	72	- 20 to + 3	97	120	+ 19
**33**	97	94	87	81	104	- 7 to + 16	79	87	+ 9
**34**	99	98	83	77	86	- 7 to + 3	116	120	+ 3
**35**	100	100	76	74	92	- 3 to + 17	70	70	+ 0
**36**	100	100	85	66	86	- 12 to + 1	73	86	+ 15

SUPPLEMENTAL FILE #4. SETUP FOR AWAKE FLEXIBLE SCOPE INTUBATION

Equipment Setup for Awake Flexible Scope Intubation (AFSI)

1. 5.5 mm flexible intubating scope (FIS) (Storz® 11301BNXK, Karl Storz SE & Co. KG, El Segundo, CA, USA) with suction connector
2. Difficult airway cart
3. 6.5 – 8.0 mm internal diameter evacuation (i.e., ‘EVAC) endotracheal tube (ETT)
4. Assorted supraglottic airway types and sizes for rescue oxygenation and ventilation
5. 4% Lidocaine 50 ml bottle: 10 ml syringe drawn up (< 9 mg/kg lean or adjusted body weight)
6. MADgic® laryngo-tracheal mucosal atomization device (MAD600, Teleflex, Morrisville, NC, USA) attached to 10 ml syringe
7. 5% Lidocaine ointment tube
8. Ovassapian intubating oral airway (IOA) (Hudson RCI® Ovassapian Fiber Optic Intubating Airway, Teleflex, Morrisville, NC, USA)
9. Water-based jelly lubricant
10. Defogger for FIS
11. Two, 6’ suction tubing or one, 10’ suction tubing with connector
12. Two, oxygen (O_2) hoses with connector
13. Three, 10 ml syringe with luer-lock for topicalization through FIS
14. Bronchoscopy 90-degree elbow adaptor
15. Double O_2 Flowmeter (with 50 psi take-off)
16. O_2 flow set to 4 L/min through the working channel of the FIS
17. Self-inflating bag with O_2 hookup and ability to deliver inhaled pulmonary vasodilators (i.e., Nitric Oxide)
18. High-Flow Oxygen Therapy (i.e., High-Flow Nasal Cannula) with appropriately sized nasal cannula
19. If requiring Bilevel Positive Pressure Ventilation (BIPAP) or Continuous Positive Airway Pressure (CPAP) – Replace with modified Patil (Endoscopy Mask 30-40-555, VBM Medizintechnik GmbH, Sulz am Neckar, Germany) with fastening straps for AFSI
20. Anchorfast ETT holder
21. Bite block
22. Blue chucks or green towels
23. N95s if required
24. Assorted syringes and blunt fill needles for intravenous (IV) drugs

<u>Drugs to Administer Pre-Intubation</u>

1. Glycopyrrolate 0.2 - 0.4 mg IV as early as possible (i.e., at least 20 minutes prior to AFSI)

Vasopressor and/or Anti-Hypertensive IV Infusions In-Line for AFSI

2. Vasopressor IV infusions (i.e., Norepinephrine, Vasopressin, Epinephrine)
3. Anti-hypertensive IV infusions (i.e., Clevidipine)
4. 'Push-Dose' syringes of vasopressors and anti-hypertensives available

Post-Intubation Drug Infusions

1. Hydromorphone IV infusion
2. Dexmedetomidine IV infusion
3. Propofol IV infusion

Drugs for Standard 'Asleep' Intubation in Case of AFSI Failure

1. One, Etomidate 10 ml (2 mg/ml) syringe
2. One, Rocuronium 10 ml (10 mg/ml) syringe
3. One, Fentanyl 3 ml (50 ug/ml) syringe
4. One, Epinephrine 10 ml (10 ug/ml) syringe
5. One, Phenylephrine 10 ml (100 ug/ml) syringe

Respiratory Therapist (RT) Role

- Avoid using the words; 'Awake Fiberoptic Intubation' to patient
- Watch all your words; to the patient, to everyone else
- Assist with holding Ovassapian IOA (Hudson RCI® Ovassapian Fiber Optic Intubating Airway, Teleflex, Morrisville, NC, USA) in place; midline
- If patient is on BIPAP: Assist with holding the Patil mask in place
    - Be sure that we have the appropriate connector for the BIPAP hose to fit on the Patil mask
    - While holding the Patil mask to get a good fit, be sure to provide some chin lift or jaw displacement so mask pressure doesn't obstruct airway
    - After IOA is placed in mouth, be sure that pressure on mask does not push on the IOA and cause discomfort
    - ETT connector will not fit through the Patil mask, so it should be removed before ETT loaded onto scope
- Extend neck and provide jaw displacement as needed for space behind the base of the tongue
- During 'railroading' procedure, observe and notify MD if FIS is advanced past carina
- After intubation, confirm verbally ETT distance at the teeth
- After intubation, initiate ventilation using low pressure settings (i.e., pressure support ventilation/positive end-expiratory pressure (PSV/PEEP) 5/5 cmH_2O) if not previously on non-invasive positive pressure ventilation (NIPPV)
- If the patient requires NIPPV (i.e., BIPAP) prior to AFSI, after intubation, initiate ventilation with the same pressure settings set on NIPPV
- Inflate ETT cuff after start of ventilation to minimize airway stimulation
- Attach bronchoscopy adaptor and use the FIS to confirm the position of the ETT

- Secure ETT with Anchorfast ETT holder
- Suction saline/H_2O through FIS suction lumen, wipe down scope, handle and cable (if FIS is reusable)

Assistant's Role

- Administer additional 4% Lidocaine as needed through working port of FIS
  - disconnect O_2 hose
  - attach Lidocaine syringe
  - administer 1 – 2 ml 4% Lidocaine x 3 (i.e., at the glottis, mid trachea and at the carina)
  - re-attach O_2 hose

Registered Nurse's Role

- Avoid using the words; 'Awake fiberoptic intubation'
- Monitor patient responses and communicate with the physician performing AFSI as needed
- Provide patient reassurance as needed
- Prepare hemodynamic and intubating medications, and administer if needed

AFSI Sequence of Events

- Explain, gargle 4% Lidocaine 2 – 3 ml
- Place IOA and have the patient bite down to hold it in place and/or have the RT hold it in midline, if needed
- Pulse spray 4% Lidocaine via IOA coordinated with inspiration only
- Advance FIS into the airway, at vocal cords, stop and spray 1 – 2 ml 4% Lidocaine
- Advance FIS into mid trachea, stop and spray 1 – 2 ml 4% Lidocaine
- Advance scope to just in front of carina, stop and spray 1 – 2 ml 4% Lidocaine
- 'Railroad' ETT over FIS, ETT tip pointing toward anterior commissure and advance ONLY on inspiration
  - If needed, administer Fentanyl 12.5 – 25 ug IV just before 'railroad' maneuver
- Confirm position of ETT, aiming for 2 – 3 cm above the carina
- Start PSV/PEEP on low pressure setting (i.e., 5/5 cmH_2O, or same settings as BIPAP if used) at 100% FiO_2 first, THEN inflate ETT cuff

STOP vital signs recorder (Epiphan Pearl Nano device, Epiphan Video, Palo Alto, CA) 5 minutes post initiation of PSV
